# Supplementary material for: Experiences, needs, and preferences for follow-up after stroke perceived by people with stroke and healthcare professionals: A focus group study
Source: PLoS One. 2019 Oct 1;14(10):e0223338. doi: 10.1371/journal.pone.0223338 (PMC6772122; doi:10.1371/journal.pone.0223338)
Supplement: S1 Checklist — (DOCX) [file pone.0223338.s001.docx]

COREQ 32-item checklist

| **No. Item** | **Guide questions/description** | **Comments** |
| --- | --- | --- |
| **Domain 1: Research team and reflexivity** |  |  |
| *Personal characteristics* |  |  |
| 1. Interviewer/facilitator | Which author/s conducted the interview? | EK (p.6) |
| 2. Credentials | What were the researcher’s credentials? *e.g., PhD, MD* | 3 Occupational Therapists (1 associate professor, 1 PhD, and 1 MS, PhD student)  1 Medical doctor, (Professor) |
| 3. Occupation | What was their occupation at the time of the study? | Researchers |
| 4. Gender | Was the researcher male or female? | 4 female (p. 6-7) |
| 5. Experience and training | What experience or training did the researchers have? | > 20 years in stroke rehab, Qualitative research (p. 7) |
| *Relationship with participants* |  |  |
| 6. Relationship established | Was a relationship established  prior to study commencement? | The moderator had met some of the health professionals in work related situations. |
| 7. Participant knowledge of the interviewer | What did the participants know about the researcher? *e.g., personal goals, reasons for doing the research* | Broad outlines given |
| 8. Interviewer characteristics | What characteristics were reported about the interviewer/facilitator? *E.g.,* *bias,* *assumptions, reasons and interests in the research topic* | (p. 6-7) |
| **Domain 2: study design** |  |  |
| *Theoretical framework* |  |  |
| 9. Methodological orientation and Theory | What methodological orientation was stated to underpin the study? *e.g. grounded theory, discourse* *analysis, ethnography,* *phenomenology, content analysis* | Focus group according to Kreuger, underpinned by a methodology based on Social constructivism (p. 4) |
| *Participant selection* |  |  |
| 10. Sampling | How were participants selected? *e.g. purposive, convenience,* *consecutive, snowball* | Purposive (p. 5) |
| 11. Method of approach | How were participants approached? e*.g. face‐to‐face,* *telephone, mail, email* | Face to face and by telephone (p. 5) |
| 12. Sample size | How many participants were in the study? | In total, 18 (patients/HPs)  participated in the focus groups (see table 2, p 9-10) |
| 13. Non-participation | How many people refused to participate or dropped out? Reasons? | One dropped out because of a medical condition (p. 9) |
| *Setting* |  |  |
| 14. Setting of data collection | Where was the data collected? *e.g. home, clinic, workplace* | Clinical setting  (p. 5 ) |
| 15. Presence of non-participants | Was anyone else present besides the participants and researchers? | No (p. 6 ) |
| 16. Description of sample | What are the important characteristics of the sample? *e.g.* *demographic data, date* | People with stroke in a late phase of stroke recovery (p. 9, table 2) |
| *Data collection* |  |  |
| 17. Interview guide | Were questions, prompts, guides provided by the authors? Was it pilot tested? | A interview guide was provided (p.6). A pilot testing was conducted. |
| 18. Repeat interviews | Were repeat interviews carried out? | No, each group met once (p. 6) |
| 19. Audio/visual recording | Did the researchers use audio or visual recording to collect the data? | Audio (p. 6 ) |
| 20. Field notes | Were field notes made during and/or after the interview or focus group? | Yes |
| 21. Duration | What was the duration of the  interviews or focus group? | Approximately 1.5 hours (p. 6 ) |
| 22 Data saturation | Was data saturation discussed? | Yes (p. 7) |
| 23. Transcripts returned | Were transcripts returned to participants for comment and/or correction? | No |
| **Domain 3: analysis and findings** |  |  |
| *Data analysis* |  |  |
| 24. Number of data coders | How many data coders coded the data? | Two (EK and GC) (p. 7 ) |
| 25. Description of the coding tree | Did authors provide a description of the coding tree? | Yes. Table 1, (p 7). |
| 26. Derivation of themes | Were themes identified in advance  or derived from the data? | Derived from the data (p. 6) |
| 27. Software | What software, if applicable, was used to manage the data? | Nvivo (p. 6) |
| 28. Participant checking | Did participants provide feedback on the findings? | No, not specifically, but a general presentation at a Stroke Association meeting. |
| *Reporting* |  |  |
| 29. Quotations presented | Were participant quotations presented to illustrate the themes/findings? Was each quotation identified? *e.g. participant number* | Yes (Results).  Each group was identified and represented. |
| 30. Data and findings consistent | Was there consistency between the data presented and the findings? | Yes (Results and Discussion) |
| 31. Clarity of major themes | Were major themes clearly presented in the findings? | Yes (Figure 3 and results) |
| 32. Clarity of minor themes | Is there a description of diverse cases or discussion of minor themes? | Yes (Results and Discussion) |
